# Attention

**Published:** 1874-11

**Authors:** 


### ATTENTION.

In this number of the Register is published an address by Dr. Allport, before the American Academy of Dental Science, at Boston. The doctor in his address takes a very decided stand upon a subject of very great importance to the profession. One about which something has occasionally been said, but it has hitherto received no extended consideration and discussion.

This matter is the separation of the mechanical department of dental practice from the medical and surgical or operative. The views presented in this address are clear and strongly stated, and are entitled to careful considerations. We will be glad to publish well digested thoughts upon the subject by any who may present them.

